# Supplementary material for: Unveiling a New Perspective on Distinguishing Omicron Breakthrough Cases and Postimmune COVID-19-Naive Individuals: Insights from Antibody Profiles
Source: Microbiol Spectr. 2023 Jul 11;11(4):e01808-23. doi: 10.1128/spectrum.01808-23 (PMC10433813; doi:10.1128/spectrum.01808-23)
Supplement: Supplemental file 1 — Table S1-Table S4.. Download spectrum.01808-23-s0001.docx, DOCX file, 0.03 MB [file spectrum.01808-23-s0001.docx]

**Supplemental Tables:**

**Table S1:** Summary of information on IgG antibody levels in 46 patients before and after two blood draws.

| ID | Blood collection time 1  (days after symptom onset) | Blood collection time 2  (days after symptom onset) | Interval between blood collections (days) | IgG-acute | IgG-recovery | IgG-recovery/IgG-acute |
| --- | --- | --- | --- | --- | --- | --- |
| 1 | 4 | 19 | 15 | 0.06 | 52.93 | 882.17 |
| 2 | 2 | 18 | 16 | 0.26 | 81.66 | 314.08 |
| 3 | 0 | 28 | 28 | 0.28 | 3.34 | 11.93 |
| 4 | 3 | 17 | 14 | 0.45 | 43.39 | 96.42 |
| 5 | 3 | 20 | 17 | 0.57 | 79.82 | 140.04 |
| 6 | 0 | 15 | 15 | 0.6 | 82.16 | 136.93 |
| 7 | 4 | 20 | 16 | 0.63 | 9.32 | 14.79 |
| 8 | 0 | 14 | 14 | 1.15 | 119.12 | 103.58 |
| 9 | 4 | 20 | 16 | 2.22 | 141.01 | 63.52 |
| 10 | 2 | 16 | 14 | 2.36 | 52.89 | 22.41 |
| 11 | 4 | 21 | 17 | 3.45 | 0.54 | 0.16 |
| 12 | 0 | 14 | 14 | 3.92 | 141.35 | 36.06 |
| 13 | 1 | 16 | 15 | 5.14 | 63.87 | 12.43 |
| 14 | 5 | 21 | 16 | 6.79 | 86.68 | 12.77 |
| 15 | 3 | 19 | 16 | 7.12 | 94.45 | 13.27 |
| 16 | 4 | 27 | 23 | 8.46 | 72.74 | 8.60 |
| 17 | 0 | 14 | 14 | 9.12 | 130.62 | 14.32 |
| 18 | 3 | 19 | 16 | 10.69 | 112.42 | 10.52 |
| 19 | 0 | 15 | 15 | 12.32 | 56.25 | 4.57 |
| 20 | 0 | 14 | 14 | 14.58 | 87.63 | 6.01 |
| 21 | 3 | 19 | 16 | 16.83 | 104.41 | 6.20 |
| 22 | 5 | 21 | 16 | 19.21 | 84.89 | 4.42 |
| 23 | 2 | 18 | 16 | 20.06 | 122.11 | 6.09 |
| 24 | 4 | 20 | 16 | 22.76 | 49.21 | 2.16 |
| 25 | 3 | 19 | 16 | 23.63 | 91.13 | 3.86 |
| 26 | 5 | 19 | 14 | 24.2 | 100.34 | 4.15 |
| 27 | 5 | 28 | 23 | 25.5 | 89.53 | 3.51 |
| 28 | 4 | 18 | 14 | 26.91 | 93.67 | 3.48 |
| 29 | 4 | 19 | 15 | 27.94 | 83.88 | 3.00 |
| 30 | 6 | 28 | 22 | 31.4 | 67.74 | 2.16 |
| 31 | 5 | 20 | 15 | 39.08 | 105.21 | 2.69 |
| 32 | 5 | 20 | 15 | 42.13 | 103.11 | 2.45 |
| 33 | 1 | 15 | 14 | 46.97 | 104.64 | 2.23 |
| 34 | 1 | 15 | 14 | 48.05 | 82.68 | 1.72 |
| 35 | 4 | 28 | 24 | 48.9 | 126.03 | 2.58 |
| 36 | 7 | 22 | 15 | 50.34 | 125.05 | 2.48 |
| 37 | 7 | 28 | 21 | 58.82 | 119.16 | 2.03 |
| 38 | 5 | 28 | 23 | 63.78 | 81.61 | 1.28 |
| 39 | 4 | 20 | 16 | 65.65 | 120.17 | 1.83 |
| 40 | 7 | 28 | 21 | 73.08 | 96.95 | 1.33 |
| 41 | 0 | 14 | 14 | 75.01 | 106.36 | 1.42 |
| 42 | 6 | 28 | 22 | 93.78 | 117.15 | 1.25 |
| 43 | 5 | 28 | 23 | 109.94 | 119.58 | 1.09 |
| 44 | 7 | 28 | 21 | 116.85 | 118.76 | 1.02 |
| 45 | 7 | 28 | 21 | 125.59 | 139.27 | 1.11 |
| 46 | 7 | 28 | 21 | 127.76 | 120.06 | 0.94 |

**Table S2**: Univariate and multivariate regression analysis of IgG antibody levels in 451 Omicron breakthrough cases.

| Variable | Observed number (N) | Univariate regression | |  | Multivariate regression^b^ | |
| --- | --- | --- | --- | --- | --- | --- |
|  |  | B | P |  | B | P |
| Total | 451 |  |  |  |  |  |
| Gender |  |  |  |  |  |  |
| Male | 271 | Reference |  |  | Reference |  |
| Female | 180 | -2.002 | 0.647 |  | -2.436 | 0.481 |
| Age (ys) |  |  |  |  |  |  |
| <18 | 52 | -13.675 | **0.043** |  | -17.086 | **0.002** |
| 18-65 | 341 | Reference |  |  | Reference |  |
| >65 | 58 | 5.382 | 0.403 |  | -4.307 | 0.402 |
| Booster dose |  |  |  |  |  |  |
| Yes | 204 | 3.686 | 0.392 |  |  |  |
| No | 247 | Reference |  |  |  |  |
| Weeks of onset |  |  |  |  |  |  |
| 0 | 32 | -10.581 | 0.120 |  | -10.254 | 0.130 |
| 1 | 217 | Reference |  |  | Reference |  |
| 2 | 137 | 50.759 | **<0.001** |  | 51.365 | **<0.001** |
| 3 | 33 | 52.051 | **<0.001** |  | 55.901 | **<0.001** |
| 4 | 17 | 74.146 | **<0.001** |  | 72.404 | **<0.001** |
| 5 | 15 | 65.828 | **<0.001** |  | 64.755 | **<0.001** |

Note: dependent variable: IgG

^b^. R=0.631, R Square=0.398, Adjusted R Square=0.387, F=36.493, P<0.001.

**Table S3**: Univariate and multivariate regression analysis of IgG antibody levels in 1149 study subjects.

| Variable | Observed number (N) | Univariate regression | |  | Multivariate regression^a^ | |
| --- | --- | --- | --- | --- | --- | --- |
|  |  | B | P |  | B | P |
| Total | 1149 |  |  |  |  |  |
| Gender |  |  |  |  |  |  |
| Male | 475 | Reference |  |  | Reference |  |
| Female | 674 | -15.547 | **<0.001** |  | -2.064 | 0.182 |
| Age (ys) |  |  |  |  |  |  |
| <18 | 52 | 51.207 | **<0.001** |  | 23.417 | **<0.001** |
| 18-65 | 920 | Reference |  |  | Reference |  |
| >65 | 177 | -12.573 | **<0.001** |  | -2.892 | 0.156 |
| Booster dose |  |  |  |  |  |  |
| Yes | 206 | 38.095 | **<0.001** |  | 5.212 | **0.031** |
| No | 943 | Reference |  |  | Reference |  |
| Group |  |  |  |  |  |  |
| Patients-acute phase | 308 | 31.645 | **<0.001** |  | 25.902 | **<0.001** |
| Patients-convalescent | 148 | 83.755 | **<0.001** |  | 77.016 | **<0.001** |
| Healthy-fully vaccinated | 693 | Reference |  |  | Reference |  |

Note: dependent variable: IgG

^a^. R=0.764, R Square=0.583, Adjusted R Square=0.581, F=266.270, P<0.001.

**Table S4**: Ability to distinguish the breakthrough cases from the post-immune population by different Cutoff values for the IgA and IgG.

| Indicators | Cutoff value | Sensitivity (%) | Specificity (%) | Positive predictive value (%) | Negative predictive value (%) | AUC |
| --- | --- | --- | --- | --- | --- | --- |
| IgA | 1 | 55.48 | 93.36 | 84.62 | 76.12 | 0.7442 |
|  | 1.5 | 50.44 | 95.53 | 88.12 | 74.55 | 0.7298 |
|  | 2 | 46.05 | 96.97 | 90.91 | 73.20 | 0.7151 |
| IgG | 15 | 67.98 | 93.22 | 86.83 | 81.57 | 0.8060 |
|  | 20 | 64.91 | 95.67 | 90.80 | 80.56 | 0.8029 |
|  | 30 | 56.58 | 98.85 | 96.99 | 77.58 | 0.7771 |
